# Supplementary material for: Chromothripsis during telomere crisis is independent of NHEJ, and consistent with a replicative origin
Source: Genome Res. 2019 May;29(5):737–49. doi: 10.1101/gr.240705.118 (PMC6499312; doi:10.1101/gr.240705.118)
Supplement: Supplemental Material [file supp_gr.240705.118_Supplemental_file_1.zip › contigs/annotated_contigs/DB87/contig.3.DB87_length_716_mean_cov_12.1103351955.docx]

**DB87_length_716_mean_cov_12.1103351955**

```
TCCAGCAATCAAGAAACAAAATATATTCTCAGCACTGGGGTCTTGACCCGACACTCACTCCCTCCCTGCCCATGTGATTAAATTTGTCT
 >chr3:100944953-100945079 + E=5e-64
TTAGATTAATGGGAATCTTCTAAACTTTATATATAT|A|CTGCTCTCCAGCCTGGGCAACAGAGCAAGACCCCATCTCTTTAAAAAAAA
                                     >chr1:31737797-31738029 - E=9e-128
ACAAAATCACATGTGAAAGCGTGGTATGAGGAAAGCCTATCCCTCTCTACCTGCTGGGGGTTGGCAAGATTTGACAGGCCACCTCACCA

ATTGGCCCCAAGTCAAGAAGCAGCTGTGTTACATAAGGCTTCTGGTTCCTGCCAAATGTTGCCATGCCAAAGTAAGGAGAGGGCAAGGA

ACC|TATATATGCATTCATCCTGAAAACTAAGAAAAAAAGGATATTAACATAGTTAACATAACAATAACATGGTCTTGATTAATATGCT
     >chr3:100945117-100945476 + E=3e-198
CAGGAAATGCAAGACACTGATAGAAGCATAAAAATACAACCCTAATTCTATCCTGCAGCAAAAAAGTTTGCTTTTTCTTTAGCAGTTTG

ATCAAAAAAAAGAAAGAATTTTATTTAGATGAATGCCTGATTATAAACTATCCATTAAGACTATTTCAAAGGTTTCCCCTTCTACCCAA

CAATAACAAATATGCTCCAAAATATTCCCTTACTAAGCCAGACTTCTCCATGTAAGAGTCTCACCTTATTTTCCTATTGAAAAACCCGA

GGCACAG
```
